# What Is the Role of Nutritional Supplements in Support of Total Hip Replacement and Total Knee Replacement Surgeries? A Systematic Review

**DOI:** 10.3390/nu10070820

**Published:** 2018-06-25

**Authors:** Louise C. Burgess, Stuart M. Phillips, Thomas W. Wainwright

**Affiliations:** 1 Orthopaedic Research Institute, Bournemouth University, 6th Floor, Executive Business Centre, 89 Holdenhurst Road, Bournemouth BH8 8EB, UK; lburgess@bournemouth.ac.uk; 2 Department of Kinesiology, McMaster University, Hamilton, ON L8S 4L8, Canada; phillis@mcmaster.ca

**Keywords:** nutrition, total hip replacement, total knee replacement, enhanced recovery after surgery, orthopedics

## Abstract

Nutritional supplements can influence outcomes for individuals undergoing major surgery, particularly in older persons whose functional reserve is limited. Accelerating recovery from total hip replacement (THR) and total knee replacement (TKR) may offer significant benefits. Therefore, we explored the role of nutritional supplements in improving recovery following THR and TKR. A systematic review was conducted to source randomized clinical trials that tested nutritional supplements in cohorts of THR or TKR patients. Our search yielded nine relevant trials. Intake of a carbohydrate-containing fluid is reported to improve insulin-like growth factor levels, reduce hunger, nausea, and length of stay, and attenuate the decrease in whole-body insulin sensitivity and endogenous glucose release. Amino acid supplementation is reported to reduce muscle atrophy and accelerate return of functional mobility. One paper reported a suppressive effect of beta-hydroxy beta-methylbutyrate, l-arginine, and l-glutamine supplementation on muscle strength loss following TKR. There is limited evidence for nutritional supplementation in THR and TKR pathways; however, the low risk profile and potential benefits to adjunctive treatment methods, such as exercise programs, suggest nutritional supplements may have a role. Optimizing nutritional status pre-operatively may help manage the surgical stress response, with a particular benefit for undernourished, frail, or elderly individuals.

## 1. Introduction

With aging, nutrition plays a significant role in maintaining physical and cognitive abilities [1,2]. Similarly, nutritional status can influence post-operative outcomes for patients undergoing major surgery, particularly in older persons whose functional reserve is limited [3]. Surgery is a stressful stimulus that results in a pro-catabolic hormonal and cytokine environment, which significantly impacts metabolism. This metabolic response is accompanied by high levels of oxidative stress, hyperinflammation, and immune system impairment, increasing the risk of post-operative complications [4]. Nutritional supplements are aimed at providing extra or absent nutrients or nutraceutical compounds either extracted from food sources or created synthetically. Nutritional supplements are wide-ranging, and specific nutritional substrates can stimulate faster recovery by modulating the metabolic response after surgery [5]. In addition, optimizing nutritional intake prior to surgery can be helpful for adjunctive treatment strategies, such as exercise, to be fully effective [6].

Often as a result of pre-surgery pain-related immobilization, orthopedic patients can suffer muscle disuse atrophy. It was suggested that increasing protein intake for older adults may preserve lean body mass and function [7]. Loading activities, such as resistance training, lead to improvements in muscle mass, strength, and function, which are enhanced by certain foods, nutrients, or nutritional supplements [8]. Essential amino acids are reported to increase the body’s capacity to synthesize protein and repair muscle tissue [9], which may allow patients a greater functional reserve to better withstand the stresses of surgery.

An important aim of Enhanced Recovery after Surgery (ERAS) is to minimize negative metabolic effects by preparing for, and reducing, the level of catabolic response post-surgery. A broad aim of ERAS is to facilitate patients’ return to a normal metabolic state as quickly as possible to facilitate recovery. Nutrition can support the reversal of surgical trauma-induced catabolism toward anabolism [5], and general surgical ERAS pathways challenged the conventional practice of pre-surgical fasting. Traditionally, pre-operative fasting is encouraged in the hours before major surgery to decrease the volume and acidity of stomach contents, thus reducing the risk of regurgitation or aspiration [10]. More recently, pre-operative carbohydrate drinks are reported to improve insulin resistance and components of patient comfort following surgery [11], and there is evidence to suggest that there is no additional benefit of prolonged pre-operative fasting [10,12]. Instead, the pathways encourage the intake of clear fluids until two hours before administering anesthesia, and a six-hour fast for solid foods [13]. Receiving a carbohydrate-rich drink prior to surgery is reported to better maintain whole-body protein balance and the suppressive effect of insulin on endogenous glucose release [14]. Avoiding pre-operative fasting, and instead, preparing the patient metabolically can reduce insulin resistance [15] and improve post-operative outcomes, as many of the metabolic changes that occur in response to surgery are due to post-traumatic insulin resistance [16]. Insulin resistance develops as a response to severe stress and following elective surgery, and the extent of the condition increases with the magnitude of surgery [15]. The failure to respond normally to the effect of insulin leads to an increase in glucose production, which when combined with a reduction in glucose uptake, can cause hyperglycemia.

Like any surgical procedure, orthopedic surgery evokes a series of stress responses [16]. Total hip replacement (THR) and total knee replacement (TKR) are two of the most common orthopedic surgeries that occur annually in the United Kingdom [17], and the aging population increases the number of procedures. Data on nutritional support for joint replacement patients are limited; however, comparable surgical studies suggest there may be additional benefit to administering nutritional supplements pre- and post-THR and TKR. As active aging increases the demand for as rapid as possible post-operative recovery and return to function, nutrition should be considered for its role in managing the surgical stress response. Therefore, our aim was to investigate the role of nutritional supplements for THR and TKR patients by completing a systematic review of the literature. Here we summarize and evaluate randomized clinical trials and pilot randomized clinical trials that investigated the role of oral nutritional supplementation in conjunction with THR and TKR surgeries.

## 2. Materials and Methods

A systematic review was conducted to examine current published evidence for the role of nutritional supplements in THR and TKR procedures. This study was developed according to the Preferred Reporting Items for Systematic Reviews and Meta-Analyses (PRISMA) statement (www.prismastatement.org/PRISMAStatement) [18].

### 2.1. Eligibility Criteria, Information Sources

A computer-based literature search was completed in November 2017, and the electronic databases reviewed included PubMed, the Cochrane Library, The Cumulative Index to Nursing and Allied Health Literature (CINAHL) Complete, and Medline Complete. The search reviewed all fields of the available peer-reviewed literature, published in the English language (or those where a translation was available) since 1990. Studies were considered eligible for inclusion within the study if they met the pre-determined inclusion and exclusion criteria (Table 1).

### 2.2. Search Strategy and Study Selection

**Figure 1:**
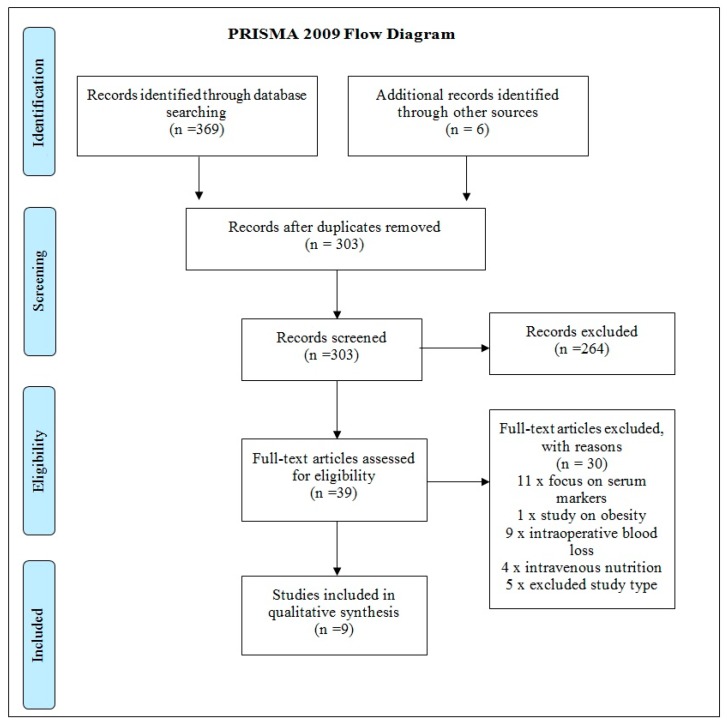
Preferred Reporting Items for Systematic Reviews and Meta-Analyses (PRISMA) flow diagram.

A search strategy was developed to capture publications that trialed oral nutritional supplements within cohorts of THR and TKR patients (Table 2). The search generated 369 results, and six additional texts were sourced from the reference lists of these papers. Once duplicates were removed, 303 texts were screened for eligibility using their titles and abstracts. Two hundred and sixty-four texts were excluded as they were irrelevant to the study. Thirty-nine articles then underwent full-text appraisal to ensure that the studies met the inclusion and exclusion criteria, and were of good methodological quality. Thirty texts were excluded, and nine texts were included in the data synthesis. A flow diagram of the study identification process can be found in Figure 1.

### 2.3. Quality Assessment

The Cochrane Collaboration’s tool for assessing risk of bias in randomized trials was used to evaluate the methodological quality of the papers included within our review. The risk-of-bias tool covers six domains of bias: selection bias, performance bias, detection bias, reporting bias, and other bias [19]. Quality assessment was not a factor for inclusion or exclusion within the systematic review, but was utilized to facilitate interpretation of findings. Two reviewers (LB and TW) completed the quality assessment, with any discrepancies resolved through discussion.

## 3. Results

The process for identifying, screening and analyzing studies is described in Figure 1 and a summary of included studies and results is shown in Table 3. 

## 4. Discussion

The results of our search (Table 3) show that, currently, there is very limited evidence upon which to make a recommendation on the use of nutritional supplements in THR and TKR surgeries. The studies included within our review mostly investigated the pre-operative intake of carbohydrate-containing fluids. The papers generally had a low risk of bias (Table S1); however, ”blinding of participants and personnel” and ”blinding of outcome assessment” were consistently reported to have a high risk of bias. A summary of all of our findings is presented below.

### 4.1. Carbohydrate Drinks

The evidence to support the pre-operative intake of a carbohydrate-rich fluid for accelerating recovery from THR and TKR is limited. It is suggested that a carbohydrate-containing drink (12.5 g carbohydrates/100 mL, pH 5.0) prior to hip replacement surgery may increase insulin-like growth factor 1 (IGF-1) levels, which plays an important role in reversing the change in metabolism following surgical stress from a catabolic state to an anabolic state [23]. Additionally, there is some evidence to suggest that a carbohydrate-containing drink may reduce hunger, nausea [24], and attenuate the decrease in whole-body insulin sensitivity [26,27] and endogenous glucose release [28]. In a pilot study [22], THR patients received a package of nutritional interventions: six hours of pre-operative fasting for solids, a drink (200 mL of 12.5% maltodextrin) up to two hours before induction of anesthesia, restricted intravenous fluids, and pre-operative immune supplementation (containing arginine, w-3 fatty acids, nucleotides and vitamins) for five days prior to surgery. The authors [22] reported that patients who received treatment had shorter hospital stays (median length of stay 3 days (range: 2–5 days) in the treatment group versus 6 days (range: 3–8 days) in the control group). Moreover, C-reactive protein, which was the secondary endpoint, when assessed during induction of anesthesia and on day two post-operatively, was lower in the intervention group (*p* < 0.01) suggesting a faster post-operative resolution of inflammation.

Intake of a pre-surgical carbohydrate-containing fluid had no significant effects on body composition after two months in physically active patients [23], and no significant effect on pain after surgery [24]. Perioperative benefits were found to be limited, and a comparison between pre-operative fasting, oral ingestion of water, or oral ingestion of a carbohydrate drink (50 kcal per 100 mL, 800 mL in the evening before surgery and 400 mL two hours before entering the operating room) found no statistically significant effect on glucose clearance, insulin sensitivity, post-operative complications, or wellbeing in patients undergoing elective hip surgery [25]. The differences in insulin sensitivity may be multifactorial, but it is difficult to make conclusions as to why due to the heterogeneity across interventions, study design, and outcome measures. Although there are no conclusive results to support the use of carbohydrate drinks for accelerating recovery, the low risk profile and potential benefits of improving post-operative insulin resistance and protein metabolism suggest they may have a role within patient care following THR and TKR. The provision of a carbohydrate-containing drink causes insulin levels to increase, which reverses a catabolic hormonal milieu, and leads to a better net-anabolic state which may help in promoting recovery post-surgery. The greatest benefit of pre-operative carbohydrate drink provision may be for elderly or frail patients, or those with multiple comorbidities, who are likely to have suboptimal recovery from surgery, and could benefit significantly from metabolic improvements.

### 4.2. Amino Acid Supplementation

Patients who received essential amino acid (EAA) supplementation (20 g) prior to TKR were reported to have reduced muscle atrophy and accelerated return of functional mobility, compared to those who received a placebo supplement [22]. The supplement was ingested twice daily between meals for one week prior to surgery and for two weeks post-operatively. Patients who received the placebo supplement demonstrated greater quadriceps muscle atrophy (−14.3 ± 3.6% change) from baseline to two weeks when compared to the EAA group (−3.4 ± 3.1%). Reduced atrophy was also observed in the non-operated quadriceps, and in the hamstrings and adductor muscles of both extremities. At two and six weeks post-operatively, the EAA group performed better on functional mobility tests (all *p* < 0.05). Twenty-eight participants completed the study (EAA = 16, placebo = 12, mean age: 69.14 ± 0.96 years), and the authors highlighted that a larger number of patients may better define the potential impact of energy and protein intake, body mass, and concomitant use of statins on the parameters of muscle mass, strength, and functional mobility following TKR.

### 4.3. Beta-Hydroxy Beta-Methylbutyrate, l-Arginine, and l-Glutamine

One paper (23 patients) outlined the suppressive effect of beta-hydroxy beta-methylbutyrate, l-arginine, and l-glutamine (HMB/Arg/Gln) on muscle strength loss following TKR, when compared to a control supplement [21]. HMB is reported to promote muscle protein synthesis and suppress muscle protein breakdown [29], and from pre-surgery to 14 days post-surgery, a significant loss of quadriceps muscle strength was reported in the control group (maximal quadriceps strength: 1.1 ± 0.62 Nm/kg before surgery and 0.7 ± 0.9 Nm/kg after surgery), but not in the HMB/Arg/Gln group (1.1 ± 0.3 Nm/kg before surgery and 0.9 ± 0.4 Nm/kg after surgery). No significant differences were observed in body weight, muscle cross-sectional area, or total energy expenditure between groups. The authors concluded that intervention with HMB/Arg/Gln nutrition and exercise may suppress the loss of muscle strength after orthopedic surgery, and lead to early improvements in physical function and fall prevention [21]. 

## 5. Future Work and Directions

Patients electing to have THR and TKR are generally in pain, have poor mobility, and suffer from muscle weakness. These complications can ultimately remain, and delay discharge post-operatively [30]. The target of nutritional therapy should, therefore, be to support those with a high risk of developing post-operative complications as a result of their surgery. We propose that sarcopenic, frail, malnourished or undernourished patients with a limited protein reserve (i.e., muscle), regardless of their baseline nutritional status, would benefit the most from perioperative nutritional support [4]. Optimizing nutritional intake prior to surgery can be helpful for adjunctive treatment strategies, such as exercise, to be fully effective [6].

Recovery from surgery is multifactorial [31], and the roles of pre- and post-operative inflammatory/immunological responses are important factors in functional recovery, pain, and post-operative fatigue [32]. The first study to characterize the inflammatory burden and muscle-specific metabolic pathways of THR patients showed that patients with a high muscle inflammatory susceptibility displayed a markedly depressed rate of mixed muscle protein synthesis in the muscle tissue surrounding the affected joint at the time of surgery, compared to those with low inflammation susceptibility [33]. According to these results [33], pre-operative local muscle inflammation susceptibility status at the time of surgery could, therefore, be an important consideration for post-operative recovery. We postulate that anti-inflammatory dietary components, such as antioxidant ingredients or n-3 polyunsaturated fatty acids (PUFAs), may help modulate key inflammatory pathways [34] and reduce pre-surgery inflammatory burden, which could accelerate recovery.

A reduced rate of muscle protein synthesis, either basally or in response to protein provision, can contribute to age-related muscle atrophy (sarcopenia) and subsequent loss of functional ability in older adults [35,36]. Muscle loss is attributed to an imbalance between muscle protein synthesis and breakdown rates, which leads to a negative muscle protein balance, and in time, a reduction in skeletal muscle mass [35]. Surgery is a catabolic insult that involves a period of muscle disuse due to bed rest. Linear sarcopenic declines in muscle mass and strength are accentuated following prolonged periods of immobility and reduced ambulation [37]. Muscle disuse following surgery is more difficult to recover from in older adults, and therefore, may lead to reductions in muscle strength and quality, exacerbation of anabolic resistance, and decrements in glycemic control [37]. Dietary consumption of protein is vital for stimulating muscle protein synthesis rates [38] and protein energy-containing supplements are more likely to have measurable impacts in older, undernourished, or malnourished individuals, and in those who are sarcopenic [7]. In addition, older adults demonstrate blunted adaptions to resistance training [39], and therefore, nutritional supplements may assist in improving adaptive responses to exercise programs.

Surgical patients that are well prepared nutritionally and physically will likely recover faster. We propose that the pre-operative period is the most effective intervention time. A combination of physical, nutritional, and psychological pre-operative preparation is reported to have beneficial roles [40]. Given their individual benefits, it appears likely that nutritional interventions combined with exercise therapy may have a greater impact than utilizing each treatment strategy individually.

Amino acid availability is an important regulator of muscle protein metabolism [41], and pre-habilitative exercise or activity may promote insulin and amino acid-sensitive muscle that can recover faster post-operatively. Intake of protein supplements, in addition to an exercise program consisting of resistive exercises, is effective for eliciting gains in fat-free mass amongst older adults [42]. There is evidence to suggest that certain foods, nutrients, or nutritional supplements can preserve lean mass in older adults [7], and enhance resistance training-induced increases in muscle mass, strength, and function [8]. For example, increases in muscle strength and lean mass in older men following six weeks of twice daily consumption of a multi-ingredient nutritional supplement (30 g whey protein, 2.5 g creatine, 500 IU vitamin D, 400 mg calcium, and 1500 mg n-3 PUFA with 700 mg as eicosapentanoic acid and 445 mg as docasahexanoic acid) are reported to be further enhanced with exercise training [43]. In addition, a significant reduction in circulating markers of systemic inflammation was observed, and these reductions were enhanced with an addition of 12 weeks of resistance training combined with high-intensity interval training [44]. Physical activity performed prior to protein intake (20 g bolus of intrinsically L-[1-(13)C]phenylalanine-labeled protein) results in greater incorporation of dietary protein into muscle [45], and resistance exercise-mediated enhancement of muscle protein synthesis to feeding persists for at least 24 h after the end of the exercise program [46].

With consideration to the findings of this systematic review, and related, supporting evidence, it is possible that the ingestion of a poly-ingredient supplement, combined with an exercise program, prior to THR and TKR may modulate the metabolic response to surgery. Future research should investigate a supplement including a high-quality protein with high leucine and anti-inflammatory dietary components, such as antioxidant ingredients or n-3 PUFAs, which may help modulate key inflammatory pathways [34]. The addition of creatine could help regenerate adenosine triphosphate which is resynthesized to release energy. In addition, HMB is a metabolite of leucine, and is thought to act as an anti-catabolic agent that minimizes protein breakdown [29]. Supplementation with HMB is reported to enhance increases in the mass and strength of skeletal muscles in elderly subjects who are recovering from bed rest [47], and may, therefore, promote recovery post-THR and TKR. Administering a carbohydrate-containing fluid pre-operatively may lead to a better net-anabolic state, most beneficial in older, frail patients, who are likely to have a suboptimal recovery from surgery. Well-conducted randomized trials with sufficient power are required to establish the benefit of combining nutritional supplements and adjunctive treatment modalities, such as an exercise program, for joint replacement patients.

## 6. Limitations

There is limited data specifically investigating the role of nutrition with THR and TKR pathways. Although of good methodological quality, the studies reported within this review had low sample sizes, and thus, the clinical significance of the results is limited. In addition, two pilot studies [22,23] were included in our review and evidence synthesis.

## 7. Conclusions

There is limited evidence for nutritional supplementation in support of patients undergoing THR and TKR; however, the low risk profile and potential benefits to adjunctive treatment methods, such as exercise programs, suggest certain supplements could play a role in enhancing recovery. Optimizing nutritional status pre-operatively may help manage the surgical stress response, and accelerate the return to function for THR and TKR patients, with a particular benefit for undernourished, frail, or elderly individuals.

## Figures and Tables

**Table 1:** Inclusion and exclusion criteria for included studies.

Inclusion Criteria	Exclusion Criteria
Population	
Total hip replacement patients	Studies on animals
Total knee replacement patients	
Intervention	
Pre-operative or post-operative oral nutrition	Intravenous nutritional supplementation Assessments of intraoperative blood loss
	Iron supplementation
Outcome Measure	
Length of stay	
Post-operative complications	
Insulin resistance	
Pain	
Functionality	
C-reactive protein	
Vitamin D	
Methodology	
Randomized clinical trials	Review articles
Pilot randomized clinical trials	Case studies
	Cross-sectional studies
	Historical studies
	Non-randomized clinical trials
Publication	
Published in English	Unpublished studies
Access to full text	Study protocols

**Table 2:** Search strategy to source texts.

Search Strategy		
Operation	Timing	Topic
(“Arthroplasty, Replacement, Hip”) OR (“Hip Prosthesis”) (Hip*) (arthroplast* OR prosthes* OR replace*) OR THA OR THR OR (“Arthroplasty, Replacement, Knee”) OR (“Knee Prosthesis”) OR (Knee*) (arthroplast* OR prosthes* OR replace*) TKA OR TKR	Preoperative OR pre-operative OR pre operative OR (“Preoperative Period”) Postoperative OR post-operative OR post operative OR (“Postoperative Period”)	Nutriti* OR (MH “Dietary Carbohydrates”) OR Carbohydrat* OR (MH “Diet+”) OR Protein OR amino acids OR “branched chain amino acid” OR Glutamine OR Omega-3 fatty acids OR Docosahexaenoic acid OR DHA OR Eicosapentenoic acid OR EPA OR Iron OR Vitamin C OR Ascorbic acid OR Vitamin D OR B vitamin* OR Selenium OR Zinc OR Calcium

THA—total hip arthroplasty; THR—total hip replacement; TKA—total knee arthroplasty; TKR—total knee replacement.

**Table 3:** Summary of studies included within review.

Study	Design and Sample Size	Patient, Population. or Problem	Intervention, Prognostic Factor or Exposure	Comparison or Intervention	Outcomes	Main Findings
Dreyer et al., 2013 [20]	RCT *n* = 28	TKR patients	20 g of essential amino acids (EAA) twice daily between meals for 1 week before and 2 weeks after TKR.	Placebo supplementation (20 g (100% alanine)	Muscle atrophy, muscle strength, and functional mobility.	The placebo group exhibited greater quadriceps muscle atrophy (−14.3 ± 3.6% change) from baseline to 2 weeks post-surgery. EAAs also attenuated atrophy in the non-operated quadriceps and in the hamstring and adductor muscles of both extremities. The EAA group demonstrated better functional mobility at 2 and 6 weeks post-operatively (all *p* < 0.05).
Nishizaki et al., 2015 [21]	RCT *n* = 23	TKR patients	Beta-hydroxy beta-methylbutyrate (HMB; 2400 mg), l-arginine (Arg; 14,000 mg) and l-glutamine (Gln; 14,000 mg) (HMB/Arg/Gln) (158 kcal of energy) for 5 days before the surgery and for 28 days after the surgery. Patients fasted on the day of surgery.	Control food (orange juice, 226 kcal of energy and 280 mg of protein)	Body weight, bilateral knee extension strength, rectus femoris cross-sectional area.	Maximal quadriceps strength was 1.1 ± 0.62 Nm/kg pre-surgery and 0.7 ± 0.9 Nm/kg 14 days post-surgery in the control group (*p =* 0.02). In the HMB/Arg/Gln group, maximum quadriceps strength was 1.1 ± 0.3 Nm/kg before surgery and 0.9 ± 0.4 Nm/kg 14 days after surgery. The muscle loss was significant in the control group, but not in the intervention group.
Alito and de Aguilar-Nascimento 2016 [22]	Pilot RCT *n* = 32	THR patients	6 h pre-operative fasting for solids, an oral drink (200 mL of 12.5% maltodextrin) up to 2 h before induction of anesthesia, restricted intravenous fluids (only 1000 mL of cystalloid fluid after surgery), and pre-operative immune nutrition (600 mL/day of Impact—Nestle, Brazil) for 5 days prior to surgery.	Control group (traditional care, 6–8 h of pre-operative fasting, intravenous hydration until the 1st post-operative day and no pre-operative immune supplementation)	Length of stay, C-reactive protein.	Median length of stay (LOS) was 3 (2–5) days in the intervention group and 6 (3–8) days in controls (*p* < 0.01). Post-operative C-reactive protein was higher in the control group (*p <* 0.001).
Aronsson et al., 2008 [23]	Pilot RCT *n* = 29	THR patients	Carbohydrate-rich drink (an iso-osmolar carbohydrate-rich solution: 12.5 g carbohydrate/100 mL, pH 5.0) pre-operatively.	Placebo drink (flavored water)	IGF-1 and IGFBP-1 were determined in serum by RIA. Body composition was determined by dual energy X-ray absorptiometry (performed the day before surgery and at 6–8 weeks after surgery)	Compared to placebo, the authors found a relative increase in IGF-1 bioavailability post-operatively after a carbohydrate-rich drink given shortly before surgery. There were no significant differences in the changes in fat or lean body mass between groups (*p* = 0.08).
Hartsen et al., 2012 [24]	RCT *n* = 60	ASA physical status I–III patients scheduled for THR	400 mL of an oral 12.5% carbohydrate solution	Placebo drink (flavored water)	Visual analog scales were used to score six discomfort parameters.	Immediately after surgery, carbohydrate-treated patients were less hungry (median scores 9.5 vs. 22 mm) and experienced less nausea (0 vs. 1.5 mm) (*p* < 0.05).
Ljunggren and Hahn 2012 [25]	RCT *n* = 57	THR patients	Tap Water: 800 mL by mouth, 2 h before entering the operating room OR Nutrition: an iso-osmolar carbohydrate drink (50 kcal/100 mL) 800 mL in the evening before the surgery (day 0), and 400 mL 2 h before entering the operating room (day 1).	Fasting (no food or water from midnight before the surgery)	Intravenous glucose tolerance, physical stress, muscle catabolism, body fluid volumes, complications, wellbeing, and insulin sensitivity.	Pre-operative ingestion of tap water or a nutritional drink had no statistically significant effect on glucose clearance, insulin sensitivity, post-operative complications, or wellbeing in patients undergoing THR.
Nygren et al., 1999 [26]	RCT *n* = 16	THR patients	Pre-operative oral iso-osmolar carbohydrate administration (800 mL 12.5% carbohydrates), the evening before the operation. Another 400 mL of the same beverage was allowed 2 h after midnight, taken no later than 2 h before the initiation of anesthesia.	Placebo drink	Insulin sensitivity	Patients given a carbohydrate drink shortly before elective surgery displayed less reduced insulin sensitivity (−16% (not significant)) after surgery compared to patients undergoing surgery after an overnight fast (37% *p <* 0.05 vs. pre-operatively). Insulin sensitivity and whole-body glucose disposal were reduced in both groups.
Soop et al., 2001 [27]	RCT *n* = 15	THR patients	Carbohydrate-rich drink (12.5 g/100 mL carbohydrate, 12% monosaccharides, 12% disaccharides, 76% polysaacharides, 285 mosmol/kg), 800 mL between 7 p.m. and 12:00 a.m. on the evening before surgery and 400 mL on the morning of surgery.	Placebo drink (acesulfame-K, 0.64 g/100 mL citrate, 107 mosmol/kg)	Glucose, lactate and insulin concentrations, glycerol, NEFA, glucoregulatory hormone concentrations, glucose kinetics, and substrate utilization.	Whole-body insulin sensitivity decreased by 18% in the intervention group vs. 43% in the placebo group. This was attributable to a less reduced glucose disposal in peripheral tissues and increased glucose oxidation rates.
Soop et al., 2004 [28]	RCT *n* = 14	THR patients	Carbohydrate rich drink (12.5 g /100 mL carbohydrate, 12% monosaccharides, 12% disaccharides, 76% polysaacharides, 285 mosm/kg, 800 mL between 7 p.m. and 12:00 a.m. on the evening before surgery and 400 mL on the morning of surgery.	Placebo drink	Glucose kinetics, substrate utilization, nitrogen balance, ambulation time, food consumption, and LOS.	Whole-body glucose disposal and nitrogen balance were similar between groups. Pre-operative carbohydrate treatment significantly attenuated post-operative endogenous glucose release (0.69 (0.07) vs. 1.21 (0.13) mg kg^−1^, (*p* < 0.01), compared to the placebo group. Whole-body glucose disposal and nitrogen balance were similar between groups.

RCT = Randomized Clinical Trial; IGF = Insulin like growth factor; IGFBP = Insulin like growth factor binding protein; RIA = Radioimmunoassay; ASA = American Society of Anesthesiologists; NEFA = nonesterified free fatty acid.

## Supplementary Materials

The following are available online at http://www.mdpi.com/2072-6643/10/7/820/s1, Table S1: Risk-of-bias assessments for included studies.
