# Supplementary material for: What Is the Role of Nutritional Supplements in Support of Total Hip Replacement and Total Knee Replacement Surgeries? A Systematic Review
Source: Nutrients. 2018 Jun 25;10(7):820. doi: 10.3390/nu10070820 (PMC6073268; doi:10.3390/nu10070820)
Supplement: Supplementary file 1 [file nutrients-10-00820-s001.pdf]

**Study**	**Bias**						
	Random sequence generation	Allocation concealment	Blinding of participants and personnel	Blinding of outcome assessment	Incomplete outcome data	Selective reporting	Other bias
Alito and de Aguilar-Nascimento 2016 [20]	+	+	−	−	+	+	+
Aronsson et al. 2008 [21]	+	+	+	−	+	+	+
Dreyer et al. 2013 [22]	+	+	+	+	+	+	−
Hartsen et al. 2012 [23]	+	+	+	−	+	+	+
Ljunggren and Hahn 2012 [24]	+	+	−	−	+	+	+
Nishizaki et al. 2015 [25]	+	−	−	−	+	+	−
Nygren et al. 1999 [26]	+	+	+	+	+	+	+
Soop et al. 2001 [27]	+	+	+	−	+	+	+
Soop et al. 2004 [28]	+	+	+	+	+	+	+

Table S1: Risk-of-bias assessments for included studies.

Key: + Low risk of bias − High risk of bias
